# Renal Tubular Dysfunction in Male Inhabitants of a Cadmium-Polluted Area in Toyama, Japan — an Eleven-year Follow-up Study

**DOI:** 10.2188/jea.11.180

**Published:** 2007-11-30

**Authors:** Yunqing Cai, Keiko Aoshima, Terutaka Katoh, Hidetoyo Teranishi, Minoru Kasuya

**Keywords:** cadmium, renal tubular dysfunction, follow-up study, urinary β_2_-microglobulin, Itai-itai disease

## Abstract

An eleven-year follow-up study was carried out to elucidate the changes in the level of environmental exposure to cadmium (Cd) from rice after soil replacement of polluted paddy fields and these effects on urinary excretion of Cd in male inhabitants of a Cd-polluted area in Toyama, Japan. In addition, the prevalence of renal tubular dysfunction (RTD) was examined to clarify the progress of Cd-induced RTD. One hundred and twenty-seven male inhabitants born between 1914 and 1929 in 11 districts of the Cd-polluted Jinzu River basin and 31 reference subjects in 2 adjacent districts were examined twice in 1985-86 and 1996-97. The geometric means of Cd concentrations in polished rice (Cd-R) in the Cd-polluted areas were 0.18 ppm in 1985 and 0.21 ppm in 1986; these values were significantly higher than those of the reference areas (0.13 ppm in 1985 and 0.12 ppm in 1986). After 11 years, the Cd-R levels were significantly decreased to approximately half (0.08 ppm in 1996, 0.12 ppm in 1997) due to soil replacement of polluted paddy fields, which has been carried out since 1980. The mean Cd levels in urine (Cd-U) were significantly reduced from 7.9 and 9.5µg/g creatinine in the initial study to 6.9 and 6.8µg/g creatinine in the follow-up study. However, the prevalence of RTD, which was determined by urinary β_2_-microglobulin exceeding 1 mg/g creatinine and urinary glucose exceeding 150 mg/g creatinine, increased from 18 and 23% in the 1985-86 study to 25 and 32% in the 1996-97 study, and a total of 12 new cases (12%) of RTD were found. Whereas, only one subject (4%) in the reference control areas was identified as RTD. Cd-induced RTD was prevalent, progressive and irreversible for male inhabitants of the Cd-polluted Jinzu River basin, although the environmental exposure to Cd through rice was significantly reduced by soil replacement of polluted paddy fields.

## INTRODUCTION

Environmental pollution with cadmium (Cd) and its effect on human health are important issues in Japan^1^^)^. Itai-itai disease, which is characterized by osteomalacia in combination with proximal tubular dysfunction, was identified in the Jinzu River basin of Toyama Prefecture, Japan, and first reported by Kohno and Hagino in 1955^2^^)^. Proximal tubular dysfunction, which is characterized by the increased urinary excretions of protein especially low-molecular weight proteins such as β_2_-microglobulin, and glucose, amino acids and enzymes, was also identified in the inhabitants of the Itai-itai disease endemic area of the Jinzu River basin^3^^-^^7^^)^. In this area, waste water containing Cd from an upstream mine had been discharged into the Jinzu River for about 50 years between the 1910’s and 1950’s^8^^)^. These circumstances had resulted in Cd pollution of the soil in the rice fields by irrigation water^9^^)^. The Prefectural Authorities carried out an extensive survey on Cd concentrations in rice and soil of the paddy fields in the Jinzu River basin over a 6-year period from 1971^10^^)^, and they declared in 1977 that the upper soil layer of a total of 1,500.6 ha of paddy fields should be replaced by non-polluted soil. An intervention program consisting of soil replacement of polluted paddy fields has been continually carried out since 1980.

By implementing a series of countermeasures against soil pollution, it has been expected that a reduction in exposure to Cd through rice may induce somewhat favorable effects on health in the inhabitants of the Jinzu River basin. However, the findings of several follow-up studies showed that Cd-induced renal tubular dysfunction is irreversible, even after reduction of exposure^11^^-^^15^^)^. Previous studies, however, mainly focused on female inhabitants and few studies on male inhabitants have been conducted concerning renal tubular dysfunction in a Cd-polluted area^12^^, ^^14^^)^. In addition, it was difficult to compare and interpret the results due to the lack of a control group. The present 11-year follow-up study for the first time aimed to compare the male inhabitants living in a Cd-polluted area with an adjacent reference area to elucidate the changes in the level of environmental exposure to Cd after soil replacement of polluted paddy fields and to clarify the prevalence and incidence of renal tubular dysfunction with urinary β_2_-microglobulin and glucose as indices.

The soil replacement project was commenced in the upper districts of the polluted Jinzu River basin in 1980 and has been continually carried out, so the duration of rice cultivation in improved rice paddies in the target areas was different between the districts. In addition, in our previous study^16^^)^, the prevalence of renal tubular dysfunction in the female population was noted to be different between the districts. Therefore, in the present study we attempted to grasp the local differences of exposure to Cd from rice and of the prevalence and incidence of renal tubular dysfunction in relation to the intervention program of soil replacement.

## SUBJECTS AND METHODS

### Research areas

**Figure 1.**
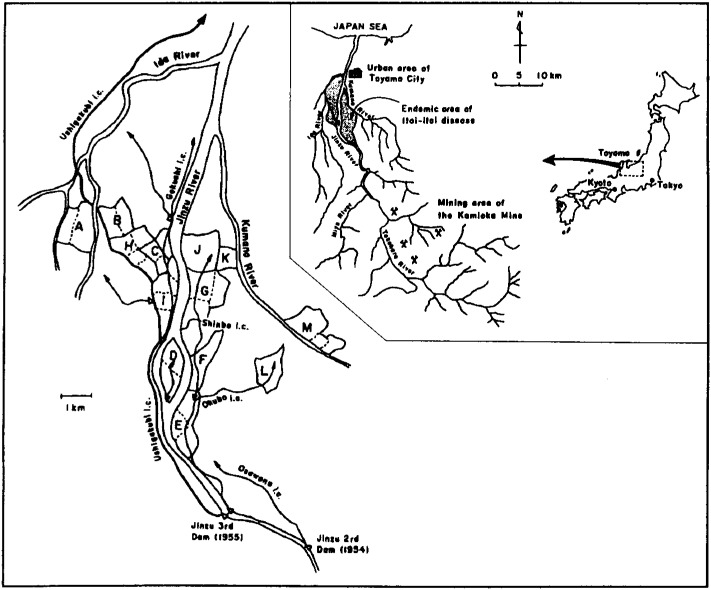
Map showing the location of the Jinzu River basin in Toyama Prefecture, Japan, and research areas. The letters B-L are cadmium-polluted districts, the letters A and M are reference districts.

Figure 1 shows the location of the research areas. Along the east-side and west-side areas of the Jinzu River basin, 11 districts, indicated by the letters B-L, including 24 hamlets, were selected. The paddy fields of these areas had been irrigated by Cd-polluted water discharged from an upstream mine through several irrigation ditches. Two districts, indicated by the letters A and M, including 5 hamlets in the adjacent Ida and Kumano River basins were selected as references areas — these 2 districts being known as free from any man-made Cd contamination — for comparison with the Cd-polluted areas in the Jinzu River basin. As stated above, the soil replacement project of a total of 1,500.6 ha of paddy fields in the Jinzu River basin including the research area has been carried out since 1980. The actual state of the soil replacement project of the research areas was previously described in detail^16^^)^.

### Subjects

The initial study was conducted in the areas A-G in 1985 and in the areas H-M in 1986. The target population was all male inhabitants born between 1918 and 1927 (58-67 years old) in the areas A-G and between 1914 and 1929 (57-72 years old) in the areas H-M. A total of 317 eligible male inhabitants in the 13 districts (A-M) were identified from the municipal population register. Of these 317 males, 140 males (89.7%) in the areas A-G and 145 (90.0%) in the areas H-L participated (Table 1). After 11 years, the follow-up study was undertaken: 111 males (79.3%) in the areas A-G and 103 (71.3%) in the areas H-M were traced. A total of 71 subjects were not traced in the follow-up examination due to the following reasons; 55 (deceased), 6 (hospitalized), 2 (moved away), 7 (refused) and 1 (disease). Of the 55 deceased subjects, 47 inhabitants of the polluted districts (B-L) died from the following reasons; malignant neoplasm (17), cerebrovascular diseases (8), cardiovascular diseases (6), respiratory diseases (4), accidents (4), digestive diseases (3), and others (5). None of them died from renal disease. Five inhabitants of the polluted area were hospitalized with cerebrovascular diseases (3), lung cancer (1) and osteoarthritis of the knee (1). Two males from the polluted target area (B-L), who were designated as suspected Itai-itai disease patients by the government of Toyama Prefecture, participated in both the initial and the follow-up studies.

**Table 1.** Distribution and characteristics of male subjects in the reference areas (A, M) and in the cadmium-polluted areas (B-G, H-L) in Toyama, Japan, in an 11-year follow-up study, 1985-86 to 1996-97.

Sampling sites	Number of participants in initial study	Number of subjects followed-up (%)		Number* of subjects studied	Age (years) at follow-up Mean±SD	Duration (years) of residence in a cadmium-polluted area at follow-up Mean±SD	Number of persons possessing rice paddies (%)		Number of persons possessing improved rice paddies (%)		Duration (years) of rice cultivation in improved rice paddies M±SD		Smoking status in initial study Current smokers / Never smokers / Former smokers	Smoking status at follow-up Current smokers / Never smokers / Former smokers
Reference area														
A	35	29	(82.9)	20	72.6±3.2	0	16	(80.0)	0	( 0 )	0		13/ 2/ 5	7/ 2/11
Cadmium-polluted area														
B	24	19	(79.2)	13	71.8±2.0	64.6±17.3	10	( 76.9)	0	( 0 )	0		5/ 4/ 4	3/ 4/ 6
C	28	19	(67.9)	15	72.1±3.0	69.5±7.8	11	( 73.3)	10	( 90.9)	1.8±1.2	[10]	8/ 1/ 5^+^	5/ 1/ 8^+^
D	10	8	(80.0)	5	72.2±2.3	67.6±11.1	5	(100.0)	5	(100.0)	4.0±2.3	[5]	3/ 0/ 2	2/ 0/ 3
E	12	11	(91.7)	9	72.7±3.5	68.3±8.9	9	(100.0)	2	( 22.2)	8.0±2.8	[2]	6/ 3/ 0	4/ 3/ 2
F	12	8	(66.7)	4	70.0±2.0	70.0±2.0	4	(100.0)	2	( 50.0)	10.5±7.8	[2]	3/ 1/ 0	2/ 1/ 1
G	19	17	(89.5)	14	72.5±3.0	70.6±3.6	13	( 92.9)	12	( 92.3)	7.6±2.4	[12]	6/ 1/ 7	4/ 1/ 9
B - G	105	82	(78.1)	60	72.1±2.7	68.4±10.1	52	( 86.7)	31	( 59.6)	5.4±3.8	[31]	31/10/18	20/10/29

Reference area														
M	22	17	(77.3)	11	75.4±3.8	0	11	(100.0)	0	( 0 )	0		4/ 6/ 1	2/ 6/ 3
Cadmium-polluted area														
H	16	11	(68.8)	8	72.4±2.9	67.6±14.3	6	( 75.0)	6	(100.0)	2.8±1.2	[6]	6/ 0/ 2	2/ 0/ 6
I	31	22	(71.0)	15	72.3±4.3	67.4±10.8	12	( 80.0)	9	( 75.0)	7.7±5.5	[9]	10/ 2/ 3	7/ 2/ 6
J	26	18	(69.2)	16	74.6±5.2	67.9±17.0	14	( 87.5)	6	( 42.9)	5.3±2.1	[6]	9/ 4/ 3	6/ 4/ 6
K	24	17	(70.8)	13	73.2±3.7	68.5±10.0	12	( 92.3)	0	( 0 )	0		8/ 4/ 1	7/ 3/ 3
L	26	18	(69.2)	15	75.0±5.4	71.3±14.0	13	( 86.7)	1	( 7.7)	16	[1]	9/ 5/ 1	6/ 5/ 4
H - L	123	86	(69.9)	67	73.6±4.6	68.6±13.2	57	( 85.1)	22	( 38.6)	6.1±4.6	[22]	42/15/10	28/14/25

* Only persons with a urinary pH above 5.50 in both 1985-86 and 1996-97 were studied.

^+^ Data not available for same one person in district C.

To evaluate the selective loss in the subjects in the follow-up study, a comparison of urinary β_2_-microglobulin or urinary glucose obtained from the initial study was made between the subjects followed-up and those lost to follow-up in each of the four areas of A, B-G, H-L and M. No significant differences in urinary β_2_-microglobulin or urinary glucose were found between the follow-up and lost-follow-up groups, except in reference area A, where the mean glucose concentrations in subjects lost to follow-up was significantly higher than that of the follow-up group (413 mg/g creatinine vs. 56 mg/g creatinine).

Among the 214 males traced, 13 subjects including 2 in the reference district M who had a history of residence in the Cd-polluted areas, 9 with diabetes mellitus and 2 with severe proteinuria not accompanied with glucosuria suggesting glomerulopathies were excluded. In addition, 43 with urinary pH < 5.50 were excluded because of degradation of β_2_-microglobulin in acidic urine^17^^, ^^18^^)^. Consequently, a total of 158 subjects (80 in the areas A-G and 78 in the areas H-M) were included in the analyses of the present study.

The characteristics of the subjects in each of the 13 districts (A-G and H-M) are summarized in Table 1. The average ages of the subjects in the 11 districts (B-L) were between 70.0 and 75.4 years old at the time of the follow-up. The average residence time for male inhabitants living in the Cd-polluted areas was 68 years at the time of the follow-up. Therefore, the inhabitants were exposed to excessive Cd for at least 50 years before soil replacement. Most subjects produced rice in their own rice paddy fields. The rates of soil improvement of the polluted paddy fields in districts C, D, G, H and I were high (75-100%) and in districts E, F and J were low (20-50%). However, in districts B, K and L the soil replacement had not been commenced by the time of the follow-up study in 1996-1997. With regard to smoking habit, the subjects were classified into current smokers, never smokers and former smokers if they had stopped smoking by the time of the initial study or the follow-up study. The rates of current smokers were lower in the follow-up study than in the initial study. A total of 34 subjects stopped smoking during the follow-up.

### Sampling and analysis

In both the initial and the follow-up studies, each subject was asked a self-administered questionnaire to obtain information about the history of residence and profession, possession and improvement of rice paddies, health status, cigarette smoking habit and use of medication. A rice sample from that consumed daily by the subject was collected. The information about the type of rice (i.e. whether the rice was produced in his-own rice field or purchased) was also obtained. Morning urine specimens were collected in polypropylene containers that were previously washed with nitric acid (1:4). Immediately after collection, the urinary pH was measured with a digital pH meter using a glass electrode. The urine samples were transferred to several tubes; the samples for β_2_-microglobulin measurement were kept above pH 6.0 by adding an ammonia solution (0.25% v/v), and for Cd determination they were kept acidified by adding nitric acid (1% v/v). All urine samples were stored at -20°C until the analysis was performed. A radio-immunoassay method was used to measure the urinary β_2_-microglobulin (β_2_-Microglobulin Radioimmunoassay Kit, Eiken Chemical Co., Ltd., Tokyo). Urinary glucose was determined by an enzymatic method (Glucose C-test Wako, Wako Pure Chemical Ind., Osaka). Creatinine analysis was performed by Jaffe’s method (Creatinine-test Wako). These measurements had been done within one month after urine collection. The same person performed the measurement of β_2_-microglobulin in urine in both the initial and the follow-up studies. The quality control for the measurement of β_2_-microglobulin was performed at the follow-up examination and involved analysis of reference serum samples with a known level of β_2_-microglobulin for each analysis day. The mean of the intra-assay precision (CV%) was 3.8±0.6% (mean±SE, N=10) in the 1996 study and 3.5±1.0% (N=11) in the 1997 study. The intra-assay precision was 7.2% at 1.48 mg/liter β_2_-microglobulin in serum in the 1996 study and 2.3% at 1.17 mg/liter β_2_-microglobulin in serum in the 1997 study. No strict quality control for the measurement of β_2_-microglobulin was done in the 1985 and 1986 study. Therefore, reanalysis for β_2_-microglobulin of the 17 urine samples from the 1986 study was performed in 1997 and there was a good correlation between the measurements in 1986 and 1997 (r=0.99, Y (1986) = 0.949X (1997) - 0.054). For the measurement of urinary glucose, daily and yearly variations were checked routinely in our laboratory.

The Japan Food Research Laboratories (Tokyo) determined the measurement of Cd in rice. The concentrations of Cd in rice were measured using an atomic absorption spectrometer after wet ashing with H_2_SO_4_/HNO_3_, and extraction was achieved with ammonium pyrrolidine dithiocarbamate and acetic acid butyl keton. Urinary Cd was measured using an atomic absorption spectrometer with Zeeman background correction (Perkin Elmer Model No. 4100ZL) equipped with a graphite furnace and AS-70 autosampler. For the determination of Cd in urine, palladium nitrate was used as the matrix modifier^19^^)^. Matrix matched calibration graphs were obtained from specimens prepared with addition of appropriate concentrations of Cd standard solutions in 0.5% nitric acid medium to urine samples with low Cd concentrations. For internal quality control, commercially available reference materials (Toxic Metals in Freeze-Dried Urine SRM2670 from the National Institute of Standard and Technology, USA) were used. The findings of the reference materials for each analysis day were within the certified values. The detection limit for Cd in urine was 0.26µg/liter. The determinations of Cd in urine and in rice obtained from the initial and the follow-up studies were performed between 1998 and 1999.

### Statistical analysis

The values of urinary β_2_-microglobulin, glucose and Cd were all adjusted for creatinine concentration. The samples with urinary creatinine concentrations ≧ 0.25 g/liter and ≦ 3 g/liter were accepted for the analysis in the present study, and all the 158 samples were accepted. For those quantities with skewed distributions, logarithmic transformations were carried out and expressed as the geometric mean (GM) and geometric-standard deviation (GSD). In view of the small numbers, the Mann-Whitney U test was used to test the statistical significance for the values between the Cd-polluted areas and the reference areas. Wilcoxon signed-ranks test for paired data was used for testing the significance of the values between the initial examination and the follow-up examination. Fisher’s exact probability test was used for testing the significance of prevalence and incidence of renal tubular dysfunction between the Cd-polluted areas and the reference areas. The level of significance was set at p<0.05. The statistical analysis was conducted using the StatView 5.0 software package.

## RESULTS

### Changes in cadmium levels in polished rice after soil replacement

Of 158 subjects studied, 145 (92%) offered rice samples in both the initial study and the follow-up study. The numbers (rates) of rice samples produced by the subjects in the areas A, B-G, M and H-L were 15 (88%), 46 (82%), 11 (100%) and 48 (79%) in the 1985-86 study, and 14 (82%), 39 (70%), 11 (100%) and 50 (82%) in the 1996-97 study, respectively. The numbers (rates) of subjects who consumed only rice produced by the subjects (the domestic rice) in both the initial and the follow-up studies were 14 (82%) in district A, 38 (68%) in districts B-G, 11 (100%) in district M and 46 (75%) in districts H-L. The remaining 36 subjects showed several variations in consuming rice between the initial and the follow-up studies, for example, from the domestic rice to the purchased rice (1 in district A, 8 in districts B-G and 2 in districts H-L), from the purchased rice to the domestic rice (1 in districts B-G and 4 in districts H-L), and only the purchased rice (2 in district A, 9 in districts B-G and 9 in districts H-L).

The averages and ranges of Cd concentrations in all of the 145 rice samples collected and only in rice samples harvested in the 13 districts (A-G) (the domestic rice) are separately shown in Table 2. Significant decreases in the concentrations of Cd in polished rice consumed daily by the participants were identified in districts C, D, G and H showing from 0.18-0.32 to 0.03-0.09 ppm. In only the domestic rice samples significant decreases in the concentrations of Cd in districts C, D, G, H and J of the Cd-polluted areas were also identified. It was interesting to note that in the follow-up study the average concentrations of Cd in domestic rice in districts C, D and G were significantly lower than that in reference district A. However, the levels of the Cd concentrations in domestic rice in districts K and L were not significantly different between the initial and the follow-up studies and were still significantly higher than that in reference district M in the follow-up study. As shown in Table 1, in districts C, D, G, H, I and J, the implementation of soil replacement was commenced between 1980 and 1996-1997. However, in districts B, K and L the implementation of soil replacement had not been commenced by 1996-1997. Therefore, these findings indicated that the replacement of the polluted soil in the paddy fields resulted in the substantial reduction of Cd intake through rice for the inhabitants of the Jinzu River basin.

**Table 2.** Cadmium levels in polished rice from male subjects in the reference areas (A, M) and cadmium-polluted areas (B-G, H-L) in Toyama, Japan, in an 11-year follow-up study, 1985-86 to 1996-97.

Site	Cadmium in total polished rice (ppm)				P*	Cadmium in domestic polished rice (ppm)				P^+^
	
	1985-86		1996-97			1985-86		1996-97		
Reference area										
A	0.13 (0.05-0.28)	[17]	0.16 (0.08-0.49)	[17]	0.120	0.13 (0.05-0.28)	[15]	0.16 (0.08-0.49)	[14]	0.183
Cadmium-polluted area										
B	0.20 (0.06-0.58)^a^	[13]	0.16 (0.05-0.88)	[13]	0.432	0.20 (0.06-0.58)^a^	[11]	0.14 (0.05-0.88)	[ 7 ]	0.222
C	0.20 (0.08-0.41)^a^	[14]	0.07 (0.01-0.18)^b^	[14]	0.001	0.27 (0.12-0.41)^a^	[ 7 ]	0.07 (0.01-0.18)^a^	[ 7 ]	0.003
D	0.32 (0.21-0.43)^b^	[ 5 ]	0.03 (0.02-0.07)^b^	[ 5 ]	0.043	0.32 (0.21-0.43)^b^	[ 5 ]	0.03 (0.02-0.07)^b^	[ 5 ]	0.009
E	0.08 (0.02-0.42)	[ 8 ]	0.09 (0.01-0.31)	[ 8 ]	0.932	0.08 (0.02-0.42)	[ 8 ]	0.09 (0.01-0.31)	[ 8 ]	0.834
F	0.14 (0.08-0.20)	[ 3 ]	0.10 (0.05-0.38)	[ 3 ]	0.593	0.14 (0.08-0.20)	[ 3 ]	0.10 (0.05-0.38)	[ 3 ]	0.513
G	0.18 (0.04-0.63)	[13]	0.09 (0.02-0.53)^b^	[13]	0.010	0.16 (0.04-0.43)	[12]	0.07 (0.02-0.13)^b^	[ 9 ]	0.043
B - G	0.18 (0.02-0.63)^a^	[56]	0.09 (0.01-0.88)^b^	[56]	<0.001	0.18 (0.02-0.58)^a^	[46]	0.08 (0.01-0.88)^b^	[39]	<0.001

Reference area										
M	0.12 (0.06-0.22)	[11]	0.09 (0.04-0.15)	[11]	0.109	0.12 (0.06-0.22)	[11]	0.09 (0.04-0.15)	[11]	0.094
Cadmium-polluted area										
H	0.21 (0.10-0.79)	[ 7 ]	0.03 (0.01-0.12)^a^	[ 7 ]	0.018	0.27 (0.10-0.79)	[ 4 ]	0.03 (0.01-0.12)	[ 5 ]	0.027
I	0.10 (0.01-1.49)	[14]	0.04 (0.01-0.27)	[14]	0.115	0.07 (0.01-0.57)	[10]	0.03 (0.01-0.27)	[11]	0.245
J	0.30 (0.09-0.86)^b^	[13]	0.13 (0.01-0.77)	[13]	0.133	0.33 (0.09-0.86)^b^	[11]	0.13 (0.01-0.77)	[12]	0.028
K	0.29 (0.10-1.14)^b^	[12]	0.29 (0.02-0.74)^b^	[12]	>0.999	0.38 (0.13-1.14)^b^	[ 9 ]	0.43 (0.23-0.74)^b^	[ 9 ]	0.691
L	0.25 (0.02-2.12)	[15]	0.25 (0.05-1.35)^a^	[15]	>0.999	0.27 (0.02-2.12)	[14]	0.25 (0.05-1.35)^a^	[13]	0.846
H - L	0.21 (0.01-2.12)^a^	[61]	0.11 (0.01-1.35)	[61]	0.011	0.23 (0.01-2.12)^a^	[48]	0.12 (0.01-1.35)	[50]	0.024

Values are geometric means with a range.

* Wilcoxon signed-ranks test for paired data between 1985 and 1996 (A to G) or 1986 and 1997 (H to M).

^+^ Mann-Whitney U test for data between 1985 and 1996 (A to G) or 1986 and 1997 (H to M).

^a^ p<0.05, ^b^ p<0.01; Significant difference compared with the reference area (A or M) (Mann-Whitney U test).

[n] Number of rice samples examined.

### Changes in urinary excretion of cadmium over 11 years

Table 3 shows the geometric means for urinary excretions of Cd in the initial and the follow-up studies in the 13 districts (A-G, H-M). In the Cd-polluted areas B-G, significant decreases in urinary excretion of Cd were found in districts C and G with significant reductions in Cd in rice after 11 years, but not in district D. In the Cd-polluted areas H-L, significant decreases in urinary Cd concentrations were found in districts K and L with no significant changes in Cd intake through rice. In reference district M, a significant decrease in urinary excretion of Cd was also found with no significant changes in Cd intake through rice. When subjects only consumed rice produced by the subjects in both the initial and the follow-up examinations were examined, similar results were obtained (data not shown).

**Table 3.** Urinary levels of cadmium, β_2_-microglobulin and glucose measured in the initial study (1985-86) and in the follow-up study (1996-97) in male subjects from the reference areas (A, M) and cadmium-polluted areas (B-G, H-L) in Toyama, Japan.

Site	Number of Subjects	Cadmium (*μ*g/g creat.)				P^+^	β_2_-microglobulin (mg/g creat.)				P^+^	Glucose (mg/g creat.)				P^+^
		
		1985-86		1996-97			1985-86		1996-97			1985-86		1996-97		
Reference area																
A	20	2.6	(2.4)	2.5	(1.8)	0.351	0.10	( 2.1)	0.22	( 3.6)	0.009	53	(1.8)	50	(1.6)	0.627
Cadmium-polluted area																
B	13	5.3	(1.6)^b^	4.9	(1.8)^b^	0.972	0.15	( 5.3)	0.45	( 4.0)	0.009	64	(3.3)	57	(1.9)	0.861
C	15	11.7	(1.5)^b^	9.4	(1.8)^b^	0.041	4.59	( 5.1)^b^	8.93	( 6.9)^b^	0.009	248	(8.1)^a^	470	(6.8)^b^	0.031
D	5	9.7	(1.9)^b^	8.6	(1.4)^b^	0.893	0.32	( 2.9)^a^	2.32	( 2.1)^b^	0.043	28	(2.3)	63	(2.2)	0.043
E	9	6.9	(3.3)^b^	9.3	(1.6)^b^	0.594	1.47	(13.2)^b^	3.15	(18.1)^a^	0.015	120	(5.9)	204	(9.7)	0.051
F	4	4.3	(1.6)	4.6	(2.3)	0.999	0.10	( 1.5)	0.15	( 2.4)	0.273	59	(2.5)	46	(1.9)	0.715
G	14	9.0	(1.7)^b^	5.9	(1.7)^b^	<0.005	1.38	( 9.1)^b^	3.66	( 7.4)^b^	0.004	76	(2.5)	215	(4.6)^b^	0.004
B - G	60	7.9	(2.0)^b^	6.9	(1.8)^b^	<0.006	0.86	( 9.7)^b^	2.22	( 9.3)^b^	<0.009	95	(4.8)	159	(5.6)^b^	<0.001

Reference area																
M	11	7.5	(1.6)	4.0	(1.5)	0.003	0.22	( 2.4)	0.30	( 3.7)	0.131	85	(1.5)	88	(1.6)	0.859
Cadmium-polluted area																
H	8	7.1	(2.4)	4.7	(2.1)	0.093	2.40	( 7.6)^b^	2.99	( 7.9)^a^	0.161	193	(4.2)	347	(5.8)	0.208
I	15	8.7	(2.1)	7.7	(1.6)^b^	0.334	0.86	(10.9)	2.38	(12.5)^a^	0.003	138	(4.4)	287	(5.9)	0.027
J	16	8.2	(1.9)	7.1	(1.5)^b^	1.163	2.76	( 7.6)^b^	4.70	( 8.4)^b^	0.070	119	(5.8)	131	(5.3)	0.438
K	13	10.4	(1.5)	5.9	(1.9)	0.002	0.49	( 6.1)	0.97	( 5.8)	0.033	121	(3.6)	143	(3.2)	0.701
L	15	13.1	(1.6)^a^	7.7	(1.8)^b^	<0.001	0.59	( 4.9)	1.30	( 6.8)	0.031	75	(1.5)	83	(2.5)	0.570
H - L	67	9.5	(1.9)	6.8	(1.8)^b^	<0.001	1.06	( 7.8)^b^	2.11	( 8.4)^b^	<0.001	118	(3.8)	161	(4.6)	0.016

Values are geometric means with geometric standard deviations in parenthesis.

^+^ Wilcoxon signed-ranks test for paired data between 1985 and 1996 (A to G) or 1986 and 1997 (H to M).

^a^ p<0.05, ^b^ p<0.01; Significant difference compared with the reference area (A or M) (Mann-Whitney U test).

Smoking is another major sources of exposure to Cd especially for males in Japan. Table 4 shows the geometric means for urinary excretion of Cd in the inhabitants of the 13 districts (A-G, H-M) according to their smoking habits at the initial and the follow-up examinations. In districts C and G, significant decreases in urinary excretion of Cd were detected in the never or former smokers, but not detected in the current smokers or the subjects who stopped smoking during the follow-up. In the Cd-polluted areas H-L, significant decreases in urinary excretion of Cd were found in the never or former smokers in district L. In addition, in districts K and L significant decreases in urinary excretion of Cd were identified in the current smokers. These findings indicated that urinary excretion of Cd might not reflect the changes in intake of Cd from rice in men in the present study.

**Table 4.** Urinary levels of cadmium (µg/g creatinine) measured in the initial study (1985-86) and in the follow-up study (1996-97) in male subjects from the reference areas (A, M) and cadmium-polluted areas (B-G, H-L) in Toyama, Japan according to smoking habits.

Site	Current smokers						Never or former						Subjects who stopped smoking during follow-up					
		
	N	1985-86		1996-97		P^+^	N	1985-86		1996-97		P^+^	N	1985-86		1996-97		P^+^
Reference area																		
A	7	2.4	(1.9)	2.0	(2.0)	0.236	7	2.1	(3.5)	2.4	(1.4)	0.735	6	3.5	(1.8)	3.2	(1.8)	0.753
Cadmium-polluted area																		
B	3	5.1	(1.8)	4.0	(1.2)^a^	>0.999	8	4.9	(1.6)	4.5	(1.9)^a^	0.886	2	7.7	(1.5)	8.9	(1.7)	0.179
C	5	9.2	(1.3)^b^	8.4	(1.6)^b^	0.345	6	11.4	(1.6)^b^	7.0	(1.3)^b^	0.046	3	16.4	(1.3)^a^	12.0	(1.2)^a^	0.108
D	2	9.0	(1.0)^a^	9.7	(1.5)^a^	0.654	2	12.9	(3.2)	8.2	(1.6)^a^	0.654	1	6.2		7.7		
E	4	10.0	(2.0)^b^	10.0	(1.8)^b^	0.715	3	8.0	(1.4)	7.0	(1.5)^a^	0.285	2	12.5	(1.7)	12.1	(1.2)^a^	0.654
F	2	3.0	(1.4)	3.5	(3.7)	0.654	1	6.4		6.5			1	5.6		5.6		
G	4	10.3	(1.4)^b^	6.4	(1.2)^b^	0.067	8	8.4	(1.6)^a^	5.2	(1.6)^a^	0.049	2	9.4	(2.8)	8.2	(2.7)	0.179
B - G	20	7.8	(1.7)^b^	6.8	(1.8)^b^	0.156	28	7.8	(1.8)^b^	5.7	(1.6)^b^	0.005	11	7.7	(3.1)^a^	9.5	(1.6)^b^	0.656

Reference area																		
M	2	10.8	(1.3)	5.2	(2.3)	0.179	7	8.2	(1.4)	4.2	(1.2)	0.018	2	3.8	(1.6)	2.7	(1.2)	0.179
Cadmium-polluted area																		
H	2	4.6	(1.9)	5.5	(3.7)	0.654	2	6.6	(1.7)	3.7	(1.7)	0.179	4	9.1	(3.0)	4.9	(2.0)	0.144
I	7	5.4	(1.9)	6.2	(1.5)	0.499	5	14.8	(1.4)^b^	9.7	(1.4)^b^	0.079	3	11.3	(2.5)	8.7	(1.7)	0.285
J	6	8.8	(1.7)	7.1	(1.6)	0.173	7	8.2	(1.4)	7.1	(1.1)^b^	0.236	3	7.0	(3.9)	7.1	(2.2)	>0.999
K	6	9.1	(1.6)	6.4	(1.9)	0.027	4	13.6	(1.3)^a^	8.0	(1.2)^b^	0.144	2	8.9	(1.8)	4.0	(2.3)	0.179
L	6	17.0	(1.4)	11.0	(1.6)	0.046	6	12.4	(1.7)	6.8	(1.8)	0.027	3	8.7	(1.5)	4.9	(1.3)	0.108
H - L	27	8.6	(1.9)	7.2	(1.8)	0.064	24	10.8	(1.6)	7.3	(1.5)^b^	<0.001	15	8.9	(2.3)	5.8	(1.8)	0.010

Values are geometric means with geometric standard deviation in parenthesis.

^+^ Wilcoxon signed-ranks test for paired data between 1985 and 1996 (A to G) or 1986 and 1997 (H to M).

^a^ p<0.05, ^b^ p<0.01; Significant difference compared with the reference area (A or M) (Mean-Whitney *U* test).

### Changes in urinary excretion of β_2_-microglobulin and glucose over 11 years

The urinary excretions of β_2_-microglobulin in the Cd-polluted areas B-G and H-L were significantly higher than in the reference area A or M in both the initial and the follow-up studies (Table 3). After 11 years, significant increases were identified in districts B, C, D, E and G, and I, K and L. In reference district A, urinary excretion of β_2_-microglobulin was also significantly increased from 0.10 to 0.22 mg/g creatinine. As shown in Table 1, although mean duration of rice cultivation in improved rice paddies in districts G and I was around 8 years, no favorable effect of reduction of Cd intake on urinary excretion of β_2_-microglobulin was found. When subjects only consumed rice produced by the subjects in both the initial and the follow-up examinations were examined, similar results were obtained.

It was also noted that urinary excretions of β_2_-microglobulin were highly correlated between the initial and the follow-up studies in the Cd-polluted areas: The Pearson correlation coefficient was 0.90 for the areas B-G, and 0.87 for the areas H-L. Age was slightly but significantly correlated with urinary excretions of β_2_-microglobulin in both the initial and the follow-up studies: The Pearson correlation coefficients in the initial and the follow-up studies were 0.37 and 0.34 for the areas B-G, and 0.39 and 0.36 for the areas H-L, respectively.

The glucose concentrations in urine in the Cd-polluted areas B-G and H-L were significantly higher in the follow-up study than those in the initial study. No favorable effect of reduction in exposure to Cd through rice on urinary excretion of glucose in districts C, D, G and I was found.

### Changes in the prevalence rates of β_2_-microglobulinuria and renal tubular dysfunction over 11 years

The prevalence of β_2_-microglobulinuria determined by exceeding 1 mg/g creatinine in each of the 13 districts (A-M) is shown in Table 5. The prevalence of β_2_-microglobulinuria was extremely high in the Cd-polluted areas, and increased from 43.3% to 63.3% in areas B-G, and from 49.3% to 58.2% in areas H-L after 11 years. The incidence rates of β_2_-microglobulinuria in the Cd-polluted areas B-G (35.3%) and H-L (29.4%) were higher than those in the reference areas A (5.0%) and M (10.0%), and significant differences were found in districts D and G compared with in district A.

**Table 5.** Prevalence and incidence of β_2_-microglobulinuria and renal tubular dysfunction (RTD) in the reference areas (A, M) and in the cadmium-polluted areas (B-G, H-L) in Toyama, Japan, in an 11-year follow-up study, 1985-86 to 1996-97.

Sampling sites	Number of subjects examined	Prevalence of β_2_-microglobulinuria				Incidence of β_2_-microglobulinuria %		Prevalence of RTD				Incidence of RTD %	
	
		1985-86 No. (%)		1996-97 No. (%)				1985-86 No. (%)		1996-97 No. (%)			
Reference area													
A	20	0	( 0 )	1	( 5.0)	5.0	( 1/20)	0	( 0 )	0	( 0 )	0	( 0/20)
Cadmium-polluted area													
B	13	2	(15.4)	5	( 38.5)*	27.3	( 3/11)	0	( 0 )	0	( 0 )	0	( 0/13)
C	15	12	(80.0)**	13	( 86.7)**	33.3	( 1/ 3)	7	(46.7)**	10	(66.7)**	37.5	( 3/ 8)*
D	5	1	(20.0)*	5	(100 )	100.0	( 4/ 4)**	0	( 0 )	1	(20.0)*	25.0	( 1/ 5)*
E	9	4	(44.4)**	5	( 55.6)**	20.0	( 1/ 5)	3	(33.3)**	3	(33.3)**	0	(0/ 6)
F	4	0	( 0 )	0	( 0 )	0.0	( 0/ 4)	0	( 0 )	0	( 0 )	0	(0/ 4)
G	14	7	(50.0)**	10	( 71.4)**	42.9	( 3/ 7)*	4	(28.6)*	5	(35.7)**	10.0	( 1/10)
B - G	60	26	(43.3)**	38	( 63.3)**	35.3	(12/34)*	14	(23.3)*	19	(31.7)**	10.9	( 5/46)

Reference area													
M	11	1	( 9.1)	2	( 18.2)	10.0	( 1/10)	0	( 0 )	1	( 9.1)	9.1	( 1/11)
Cadmium-polluted area													
H	8	5	(62.5)*	5	( 62.5)	33.3	( 1/ 3)	2	(25.0)	3	(37.5)	16.7	( 1/ 6)
I	15	6	(40.0)	8	( 53.3)	22.2	( 2/ 9)	5	(33.3)	6	(40.0)	20.0	( 2/10)
J	16	12	(75.0)**	13	( 81.3)**	50.0	( 2/ 4)	4	(25.0)	4	(25.0)	8.3	( 1/12)
K	13	4	(30.8)	5	( 38.5)	22.2	( 2/ 9)	1	( 7.7)	2	(15.4)	8.3	( 1/12)
L	15	6	(40.0)	8	( 53.3)	33.3	( 3/ 9)	0	( 0 )	2	(13.3)	13.3	( 2/15)
H - L	67	33	(49.3)*	39	( 58.2)*	29.4	(10/35)	12	(17.9)	17	(25.4)	12.7	( 7/55)

β_2_-Microglobulinuria was determined by urinary β_2_-microglobulin exceeding 1 mg/g creatinine.

Renal tubular dysfunction (RTD) was determined by urinary β_2_-microglobulin exceeding 1 mg/g creatinine and urinary glucose exceeding 150 mg/g creatinine.

*p<0.05, **p<0.01; Significant differences compared with the reference area (A or M) (Fisher’s exact probability test).

The prevalence of renal tubular dysfunction determined by β_2_-microglobulin exceeding 1 mg/g creatinine and glucose exceeding 150 mg/g creatinine is also shown in Table 5. The prevalence of renal tubular dysfunction increased from 23.3% to 31.7% in the Cd-polluted areas B-G, and from 17.9% to 25.4% in the areas H-L after 11 years. A total of 12 new cases of renal tubular dysfunction were found in the Cd-polluted areas, and one in reference area M. No cases with renal tubular dysfunction were found to be recoverable based on the value of both the urinary β_2_-microglobulin and glucose which dropped to below the cut-off value. Only two cases among 26, found in the initial study, were excluded from the prevalence in the follow-up study because their urinary glucose levels decreased to below 150 mg/g creatinine, but their urinary β_2_-microglobulin was still progressive. These findings demonstrated that the prognosis of Cd-induced renal tubular dysfunction for male subjects in the Jinzu River basin was deteriorated, and it was also possible to develop renal tubular dysfunction, even in Cd-exposed subjects without renal tubular dysfunction in the initial study.

## DISCUSSION

### Changes in exposure to cadmium from rice over 11 years

The purpose of the present study was to assess the changes in the levels of exposure to Cd from rice after soil replacement of paddy fields for the male inhabitants in the Cd-polluted Jinzu River basin. In the target areas of the present study many professional farmers and farming families had produced only rice in their paddy fields and consumed their own produced rice throughout the year. Therefore, rice samples are suitable for exposure measurement to Cd in the subjects of the present study.

Previous studies, which determined Cd concentrations in rice in the Jinzu River basin, demonstrated that there was no indication that a substantial change in Cd exposure from rice had occurred between 1967 and 1977^4^^, ^^5^^, ^^20^^)^. In our previous study^16^^)^, the mean Cd concentration in domestic rice in the Cd-polluted areas B-G was 0.27 ppm in 1983 and in areas H-L was 0.33 ppm in 1984. These values were similar to the levels determined in 1967 in the same areas^20^^)^. This suggests that Cd levels in rice had not changed between 1967 and 1984. In district I, the mean Cd level in rice in the initial study in 1986 was 0.07 ppm. In our previous study^16^^)^, however, the mean Cd level in domestic rice collected from 12 female inhabitants in district I was 0.44 ppm in 1984. Consequently, a remarkable decline in Cd levels in rice collected from district I was found between 1984 and 1986, since district I was one of the earliest sites for the replacement of polluted soil. Therefore, the implementation of soil replacement in the paddy fields was clearly very effective to reduce Cd intake through rice for inhabitants in a Cd-polluted area.

### Effects of changes in the levels of exposure to cadmium on urinary excretion of cadmium

In general, urinary excretion of Cd is considered to reflect the total body burden of Cd^1^^)^. Many studies have also suggested that urinary Cd is an indicator of Cd exposure and has a dose-response relationship with the levels of Cd in rice^21^^, ^^22^^)^. Kido et al.^22^^)^ investigated 1,815 inhabitants of the Cd-polluted Kakehashi River basin in Ishikawa Prefecture and 240 inhabitants of a reference control area; the inhabitants were divided into three groups according to the average Cd concentration in rice. In that study, the mean urinary Cd concentration was increased in a dose-related manner in inhabitants of the polluted area. In the present study, however, the examinations on a group basis of each 11 districts showed that significant decreases in urinary excretion of Cd were found in districts C and G with significant reductions in Cd in rice, and in districts K and L with no significant changes in Cd intake through rice. In reference district M, a significant decrease in urinary excretion of Cd with no significant changes in Cd intake through rice was also found. These showed that urinary Cd excretion might not reflect the changes in exposure to Cd through rice in males in the present study.

In previous findings for female inhabitants, however, significant decreases in urinary excretion of Cd were found in accordance with the degrees of changes in Cd levels in rice^16^^)^. One explanation for this discrepancy between males and females could be smoking, since smokers have been more exposed to Cd than non-smokers^23^^, ^^24^^)^. Therefore, we examined the influences of smoking on the changes in urinary excretion of Cd for 11 years (Table 4). Significant decreases in urinary excretion of Cd were identified only in the never or former smokers in the Cd-polluted areas B-G and H-L. Thus, smoking habits influenced urinary excretion of Cd in males of the present study. However, in districts K and L, significant decreases in urinary Cd were also identified in the current smokers. Although we could not fully explain these findings, several factors such as the reduction in rice intake and cigarette consumption with age might be considered^23^^, ^^25^^)^. Consequently, it appears that the Cd level in urine is not a suitable monitoring indicator for Cd exposure from rice in inhabitants of a Cd-polluted area, especially in the males.

In reference district M, urinary excretion of Cd was higher compared with that of district A in both the initial and the follow-up studies. A similar tendency was also found in women in our previous study^16^^)^. The reasons why urinary excretions were higher in reference district M could not be explained. However, one reason might be that the Kumano River originates from the range of mountains in which large-scale ore deposits rich in heavy metals including Cd exist and of which a representative is the Kamioka mine (Fig. 1). Cd concentrations in rice, however, were not different between district M and district A (Table 2).

### Changes in urinary excretion of β_2_-microglobulin and glucose and prevalence rates of renal tubular dysfunction over 11 years

Few studies have focused on the prognosis of renal tubular dysfunction in males living in Cd-polluted areas in Japan. Since Itai-itai disease is prevalent in females, attention has been given more to females than to males^4^^)^. Therefore, understanding the progress and prognosis of renal tubular dysfunction found in male inhabitants in Cd-polluted areas has been limited.

A number of studies have demonstrated that urinary β_2_-microglobulin excretion was a sensitive indicator to assess the changes in renal tubular function and was extensively used in epidemiological and occupational studies for Cd-induced renal tubular dysfunction^11^^-^^15^^, ^^26^^, ^^27^^)^. However, the use of an index for β_2_-microglobulin in urine has not taken urinary pH into consideration in previous follow-up studies^13^^-^^15^^, ^^26^^)^. Since β_2_-microglobulin is known to be unstable in urine with a pH < 5.50^17^^, ^^18^^)^, even in the bladder, misleading results may be obtained for β_2_-microglobulin evaluation without consideration of urinary pH. Only those subjects whose urinary pH was above 5.50 were included in the analyses of the present study. Cd-induced renal tubular dysfunction is also accompanied by raised urinary glucose^3^^, ^^27^^, ^^28^^)^. Thus, in the present study, β_2_-microglobulin and glucose were mainly used as indicators to assess the changes in renal tubular function.

As shown in Tables 3 and 5, the urinary β_2_-microglobulin concentrations of subjects living in the Cd-polluted areas B-G and H-L were significantly increased after 11 years. Approximately 60% of male inhabitants had β_2_-microglobulin-uria determined by urinary β_2_-microglobulin exceeding 1 mg/g creatinine at the time of the follow-up study. These findings clearly revealed that the male inhabitants studied had also been severely affected by exposure to Cd similar to females^16^^)^. Indeed, we previously reported that the male subjects with fractional excretion of β_2_-microglobulin (FE β_2_-m) exceeding 10%, who were included in the present study, were at risk for development of osteomalacia, and those with FE β_2_-m level exceeding 30% manifested generalized proximal tubular dysfunction clinically as well as females^27^^)^.

In the present study, clearly different prevalence rates of renal tubular dysfunction were found in the different areas within the Jinzu River basin in both the initial and the follow-up studies. As shown in Table 5, the prevalence rates of renal tubular dysfunction in districts C, E, G, H, I and J showed high rates ranging from 25% to 67%. In contrast, the prevalence rates in districts B, D, F, K and L showed lower levels ranging from 0% to 20%. These differences could not be explained by the levels of Cd in rice produced in each district (Table 2), or by the intervention program of soil replacement (Table 1). These findings suggest that the local situations of each district, such as in relation to the irrigation ditches, are important determinants of changes in the prevalence and incidence of renal tubular dysfunction in the inhabitants of the Jinzu River basin^6^^, ^^16^^)^.

Cd is accumulated in the kidney cortex with a biological half-time of 10-30 years^1^^)^. Consequently, even if the exposure to Cd from rice were decreased, the concentration of Cd in the kidneys would not be decreased. Therefore, it is reasonable to suggest that it appears to be difficult to evaluate the effects of reduction of exposure to Cd from rice on renal tubular dysfunction in such cases with long-term exposure to Cd. This suggests that a continuation of long-term follow-up studies, as reported here, is the most valuable means for elucidating the prognosis of renal tubular dysfunction among populations exposed to Cd, and also the processes of the development in different genders.

One limitation of the present study was to reduce the subjects examined because of the acidic urine for evaluation of β_2_-microglobulin. As previously reported^18^^)^, mean urinary pH in males was more decreased than that in females. Almost 55% of samples from males had a pH below 5.80. Therefore, a more stable index such as *α*_1_-microglobulin or N-acetyl-β-D-glucosaminidase should be used for further follow-up examinations.
